# Supplementary material for: Minute-scale control of ubiquitin-mediated degradation reveals dynamics of bacterial secreted effector-functions
Source: Nat Commun. 2026 May 18;17:4420. doi: 10.1038/s41467-026-73213-x (PMC13183884; doi:10.1038/s41467-026-73213-x)
Supplement: Supplementary file 1 — Supplementary Information [file 41467_2026_73213_MOESM1_ESM.pdf]

SUPPLEMENTARY INFORMATION

Minute-scale control of ubiquitin-mediated degradation reveals dynamics of bacterial secreted effector-functions

Haolin Zhang[1], Yongxia Guo[1], Bikash Adhikari[2], Nevenka Dudvarski-Stankovic[2], Elmar Wolf[2], Thomas Rudel[1]

[1]Chair of Microbiology, University of Wuerzburg, Wuerzburg, Germany

[2] Institute of Biochemistry, University of Kiel, Kiel, Germany.

*Correspondence

Thomas Rudel, Email: thomas.rudel@uni-wuerzburg.de

Content:

Supplementary Table 1
Supplementary Table 2
Supplementary Table 3
Supplementary Table 4
Supplementary Figures 1-17
Supplemental References

Supplementary Table 1. Cell lines used in this study

Cell line	Description	Source
McCoy	ATCC CRL-1696	[1]
HeLa 229	ATCC CCL-2.1	[2]
HEK293T	ATCC CRL-3216	[3]
A-375	ATCC CRL-1619	[4]
HCT 116	ATCC CCL-247	[5]
U-2 OS	ATCC HTB-96	[6]
PMRT	Primary Murine Reproductive Tract cells	This study
HeLa-OsTIR1(F74G)	HeLa cells stably express OsTIR1(F74G)-9x Myc	This study
HeLa-OsTIR1(E7K/E10K/F74G)	HeLa cells stably express OsTIR1(E7K/E10K/F74G)-9x Myc	This study
A-375-OsTIR1(F74G)	A-375 cells stably express OsTIR1(F74G)-9x Myc	This study
HCT 116-OsTIR1(F74G)	HCT 116 cells stably express OsTIR1(F74G)-9x Myc	This study
U-2 OS-OsTIR1(F74G)	U-2 OS cells stably express OsTIR1(F74G)-9x Myc	This study
PMRT-OsTIR1(F74G)	Primary Murine Reproductive Tract cells stably express OsTIR1(F74G)-9x Myc	This study
PMRT-OsTIR1(E7K/E10K/F74G)	Primary Murine Reproductive Tract cells stably express OsTIR1(E7K/E10K/F74G)-9x Myc	This study

Supplementary Table 2. Plasmids used in this study

Name	Description	Source
pKW-L2	Suicidal plasmid for FRAEM genome-editing	[7]
pHL18	pCMV-N1 plasmid expressing GFP-mAID-NLS-FLAG	This study
pHL20	pCMV-N1 plasmid expressing GFP-NLS-FLAG	This study
pHL54	pKW-L2-3kb upstream-Cdu1-mAID-FLAG-3kb downstream	This study
pHL55	pKW-L2-3kb upstream-Cdu1-FLAG-3kb downstream	This study
pHL92	pKW-L2-3kb upstream-IncA-mAID-FLAG-3kb downstream	This study
pHL93	pKW-L2-3kb upstream-IncA- FLAG-3kb downstream	This study
pHL130	pKW-L2-3kb upstream-IncA::SpecR-3kb downstream, IncA KO	This study
pRRL_OsTIR1F74G	Plasmid used for producing Lentivirus expressing OsTIR1(F74G)-9x Myc	This study
pRRL_OsTIR1F74G_2mut	Plasmid used for producing Lentivirus expressing OsTIR1(E7K/E10K/F74G)-9x Myc	This study
pBOMB5-Tet-CtrR3-IncA_aadA	Plasmid used for sRNA inhibition of IncA in *Chlamydia trachomatis*	From Paul Köhling, [8]
psPAX2	Lentivirus production helper plasmid	Addgene, Cat. # 12260

pMD2.G	Lentivirus production helper plasmid	Addgene, Cat. # 12259

Supplementary Table 3. Strains used in this study

Strain	Description	Source
DH10B	F– *mcrA* Δ(*mrr-hsdRMS-mcrBC*) φ80*lacZ*ΔM15 Δ*lacX74 recA1 endA1 araD139* Δ (*ara-leu*)7697 *galU galK* λ– *rpsL*(Str^R) *nupG*	Thermo Scientific
Chlamydia trachomatis	Serovar LGV L2 (434)	ATCC® VR-902B™ [9]
eHL18	DH10B expressing pHL18	This study
eHL20	DH10B expressing pHL20	This study
eHL54	DH10B expressing pHL54	This study
eHL55	DH10B expressing pHL55	This study
eHL92	DH10B expressing pHL92	This study
eHL93	DH10B expressing pHL93	This study
eHL130	DH10B expressing pHL130	This study
cHL54	*Chlamydia trachomatis* strain expressing Cdu1-mAID-FLAG by its genome	This study
cHL55	*Chlamydia trachomatis* strain expressing Cdu1- FLAG by its genome	This study
cHL92	*Chlamydia trachomatis* strain expressing IncA-mAID-FLAG by its genome	This study
cHL93	*Chlamydia trachomatis* strain expressing IncA-FLAG by its genome	This study
cHL130	*Chlamydia trachomatis* IncA Knockout strain	This study
cHL_IncA_sRNA	*Chlamydia trachomatis* strain expressing plasmid pBOMB5-Tet-CtrR3-IncA_aadA	This study

Supplementary Table 4. Antibodies used in this study

Antibodies	Dilution	Description & Source
anti-cHSP60	1:200 (IF)<br>1:50 (ExM)	Primary antibody, Santa Cruz, #sc-57840
anti-p62	1:200 (IF)	Primary antibody, Santa Cruz, #sc-28359
anti-OmcB	1:1000 (WB)	Primary antibody, Invitrogen, #PA5-117552
anti-ompA	1:500 (WB)	Primary antibody, Invitrogen, #PA5-117609
anti-IncA	1:100 (WB)	Primary antibody, self-made
anti-Cdu1	1:100 (WB)	Primary antibody, self-made
Anti-DDDDK tag	1:500 (IF)<br>1:200 (ExM)	Primary antibody, Abcam, #ab205606
Alexa Fluor™ Plus 488	1:300 (IF)	Secondary antibody, Invitrogen, #A32723
Alexa Fluor™ Plus 555	1:300 (IF)	Secondary antibody, Invitrogen, #A32732
anti-Rabbit IgG (H+L), HRP	1:50000 (WB)	Secondary antibody, Invitrogen, #31460
anti-Mouse IgG (H+L), HRP	1:50000 (WB)	Secondary antibody, Invitrogen, #31430
anti-alpha Tubulin HRP	1:50000 (WB)	Hrp preconjugated antibodies, Abcam, #ab185067
anti-Myc HRP	1:1000 (WB)	Hrp preconjugated antibodies, Abclonal, #AE026
anti-DDDDK-Tag HRP	1:5000 (WB)	Hrp preconjugated antibodies, Abclonal, #AE095

anti-GAPDH HRP	1:25000 (WB)	Hrp preconjugated antibodies, Abclonal, #19056
anti-β-Actin	1:25000 (WB)	Hrp preconjugated antibodies, Abclonal, # AC028

[a]ExM (Expansion Microscopy), IF (Immunofluorescence), WB (Western Blot)

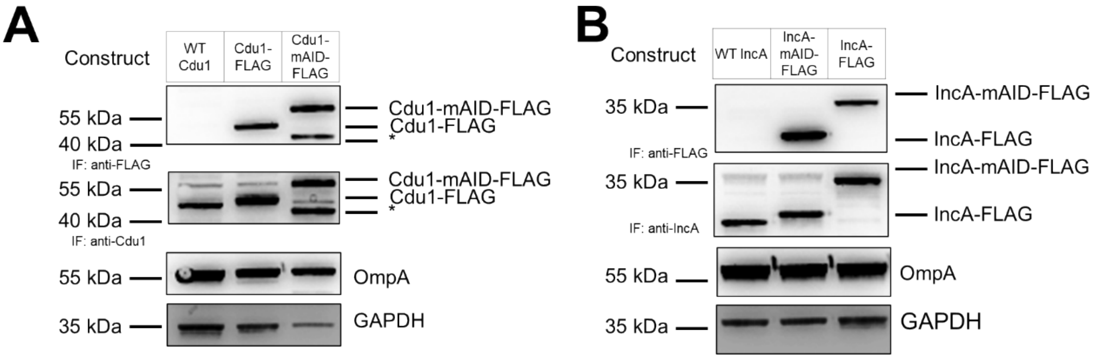

Supplementary Figure 1. (A) Immunoblot analysis of Cdu1-FLAG and Cdu1-mAID-FLAG expression in recombinant chlamydial strains. Cdu1 constructs were detected with anti-FLAG antibody and anti-Cdu1 antibody; *Chlamydia* OmpA and host cell GAPDH served as a loading control. Asterisks denote putative truncated or fragmented forms of Cdu1. (B) Immunoblot analysis of IncA-FLAG and IncA-mAID-FLAG expression in recombinant strains. IncA constructs were detected with anti-FLAG antibody and anti-IncA antibody; *Chlamydia* OmpA and host β-actin and GAPDH served as loading control. All experiments were replicated ≥3 times with consistent results. Source data are provided as a Source Data file.

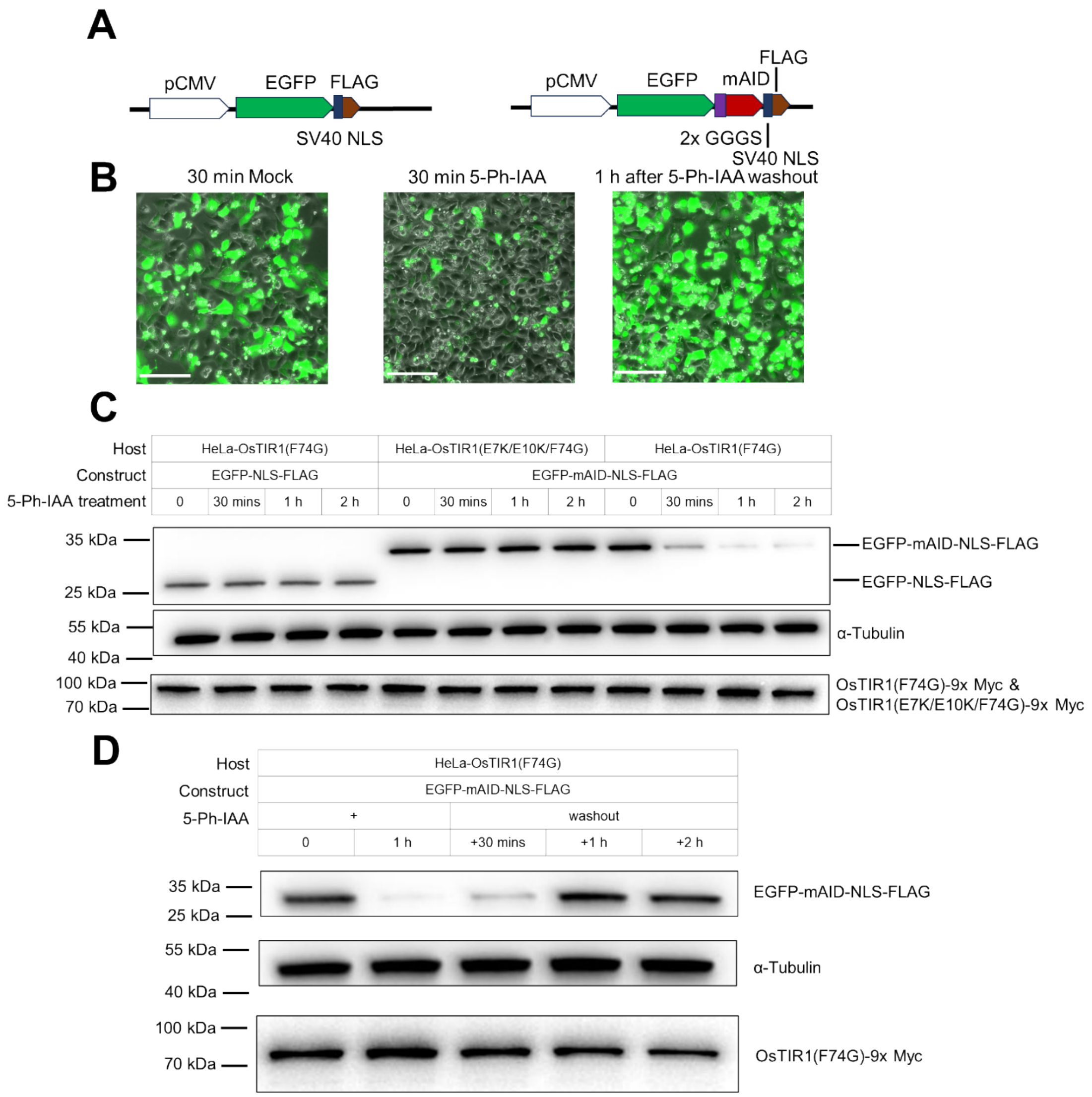

Supplementary Figure 2. Development and validation of the AID2 system for spatiotemporal control of protein abundance in HeLa cells. (A) Schematic of cassette expressing the GFP mutants used for degradation. SV40 NLS, nuclear localization signal. (B) Time-resolved GFP depletion and recovery. Live-cell microscopy of HeLa cells co-expressing OsTIR1(F74G) and mAID-GFP. GFP fluorescence diminishes within 30 minutes of 1 μM 5-Ph-IAA treatment and recovers 1 hour after auxin washout. Scale bar = 100 μm. (C) Immunoblot validation of AID2 specificity. Robust GFP degradation requires both mAID tagging and functional OsTIR1(F74G). Minimal degradation occurs with untagged GFP or catalytically impaired OsTIR1(E7K/E10K/F74G). α-tubulin serves as a loading control; OsTIR1-F74G expression was detected using anti-Myc antibody. (D) Reversibility of AID2-mediated degradation. Cells were treated with 1 μM 5-Ph-IAA for 30 minutes, after which auxin was removed and GFP levels were assessed at the indicated recovery time points. GFP levels rebound within 1 hour of auxin removal, demonstrating dynamic

control. All experiments were replicated ≥3 times with consistent results. Source data are provided as a Source Data file.

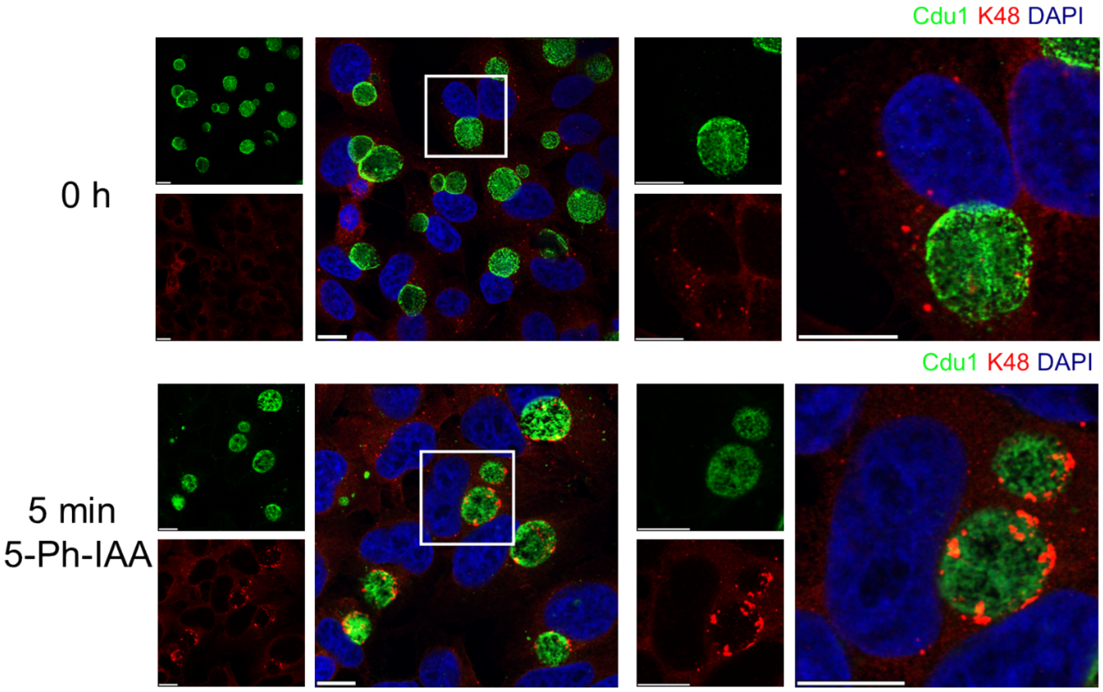

Supplementary Figure 3. Ctr-AIDE induces rapid K48-ubiquitin conjugation to Cdu1 during degradation. Immunofluorescence microscopy reveals K48-ubiquitin recruitment to the inclusion membrane within 5 minutes of auxin treatment. The rapid K48-ubiquitin signal reflecting host-mediated tagging of Cdu1-mAID during degradation, may interfere with the downstream signal caused by inclusion ubiquitination due to Cdu1 degradation. Cdu1 (green, anti-FLAG) was visualized with K48-Ubiquitin (red), and DAPI (blue) marking bacterial and host DNA. The white square in the left panel marks the region enlarged in the right panel. Scale bar = 10 µm. All experiments were replicated ≥3 times with consistent results.

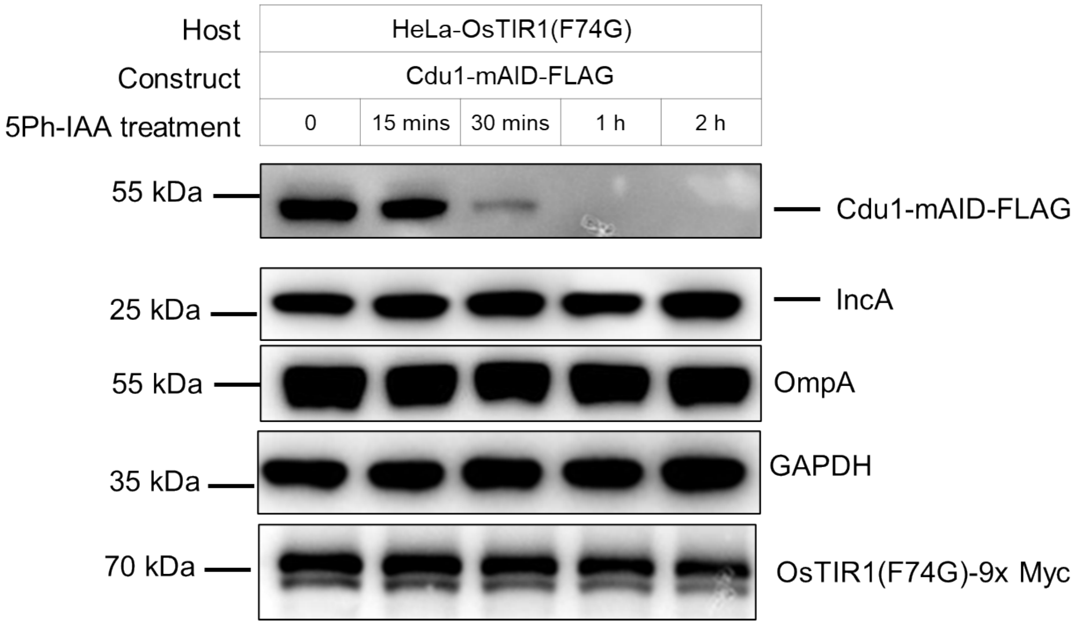

Supplementary Figure 4. Ctr-AIDE selectively degrades mAID tagged effector. HeLa cells expressing OsTIR1(F47G) were infected with Cdu1-mAID expressing strains at MOI=1. Cdu1 (anti-FLAG), IncA (anti-IncA) and OsTIR1 mutants (anti-Myc) levels were monitored, with Chlamydial Major Outer Membrane Protein (OmpA) as a loading control and GAPDH as a host cell control. All experiments were replicated ≥3 times with consistent results. Source data are provided as a Source Data file.

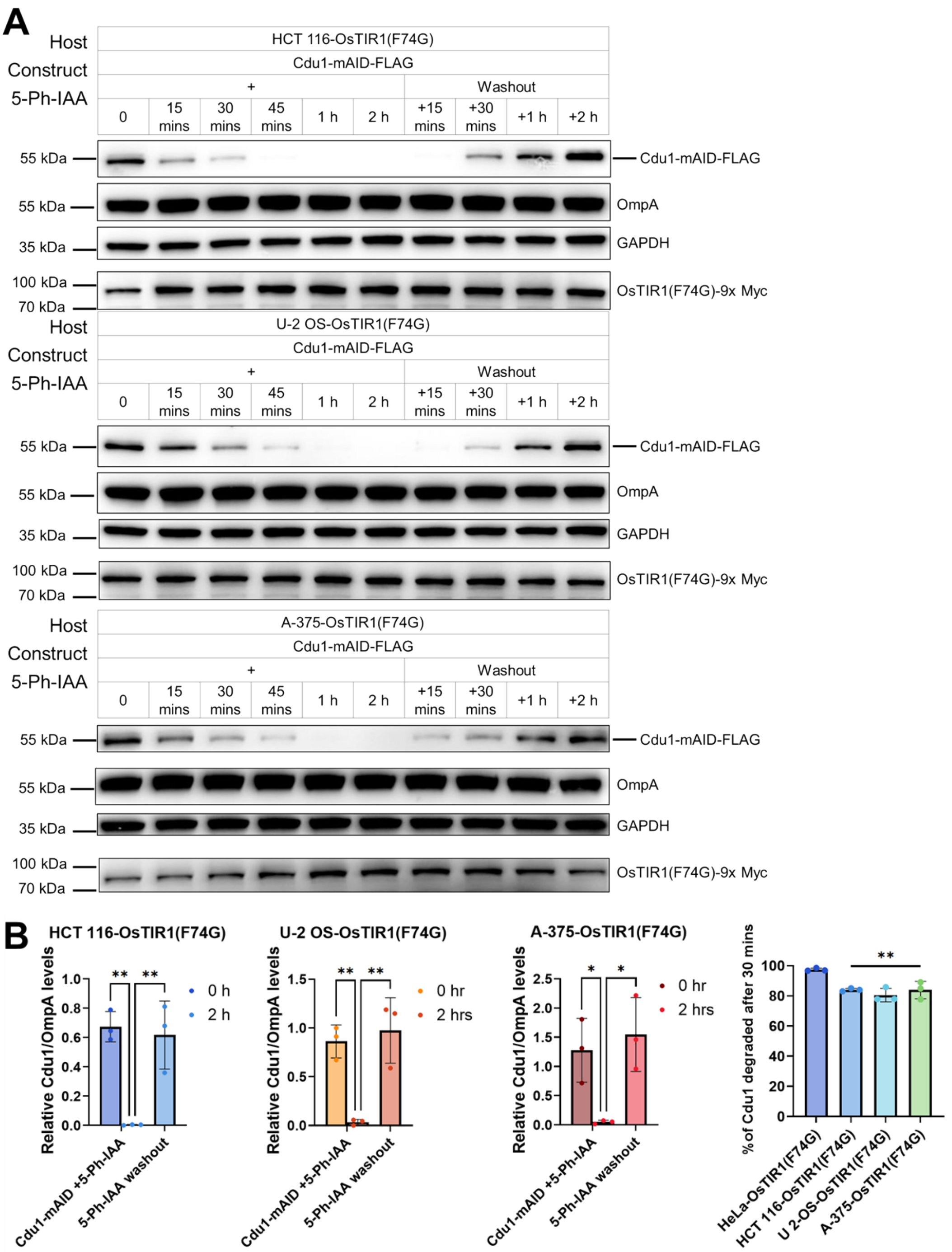

Supplementary Figure 5. Ctr-AIDE-mediated Cdu1 degradation in various cancer cell lines. (A) Ctr-AIDE-mediated regulation of Cdu1-mAID expression in different cancer cell lines. A-375, HCT 116 and U-2 OS cells expressing OsTIR1(F74G) were infected with Cdu1-mAID expressing *Chlamydia* (MOI = 1). Cells were treated with 1 μM 5-Ph-IAA for 2 hours (degradation) or further washed and incubated in 5-Ph-IAA free medium for 2 hours (recovery). Cdu1 (anti-FLAG) and OsTIR1(F74G) (anti-Myc) levels were monitored, with *Chlamydia* OmpA as a loading control and host GAPDH as a cellular control. (B) Quantification and comparation of Cdu1 degradation efficiency in various cancer cell lines. Quantification (left three panels) shows Cdu1-

FLAG levels normalized to OmpA (mean ± SD; n = 3 biological replicates). Significance assessed by two-tailed paired t-test (**$p < 0.01$; *$p < 0.05$; n.s., not significant, exact p values were provided in Supplementary Data 3). Comparation (right panel) shows relative decrease of Cdu1 levels after 30 minutes of 1 μM 5-Ph-IAA treatment (normalized to 0-hour controls). Data represent mean percentage decrease ± SD (n = 3 biological replicates; one representative blot in Panel A. Significance versus HeLa: **$p < 0.01$ (one-way ANOVA with Tukey Multiple comparisons test, exact p values were provided in Supplementary Data 3). All experiments were replicated ≥3 times with consistent results. Source data are provided as a Source Data file.

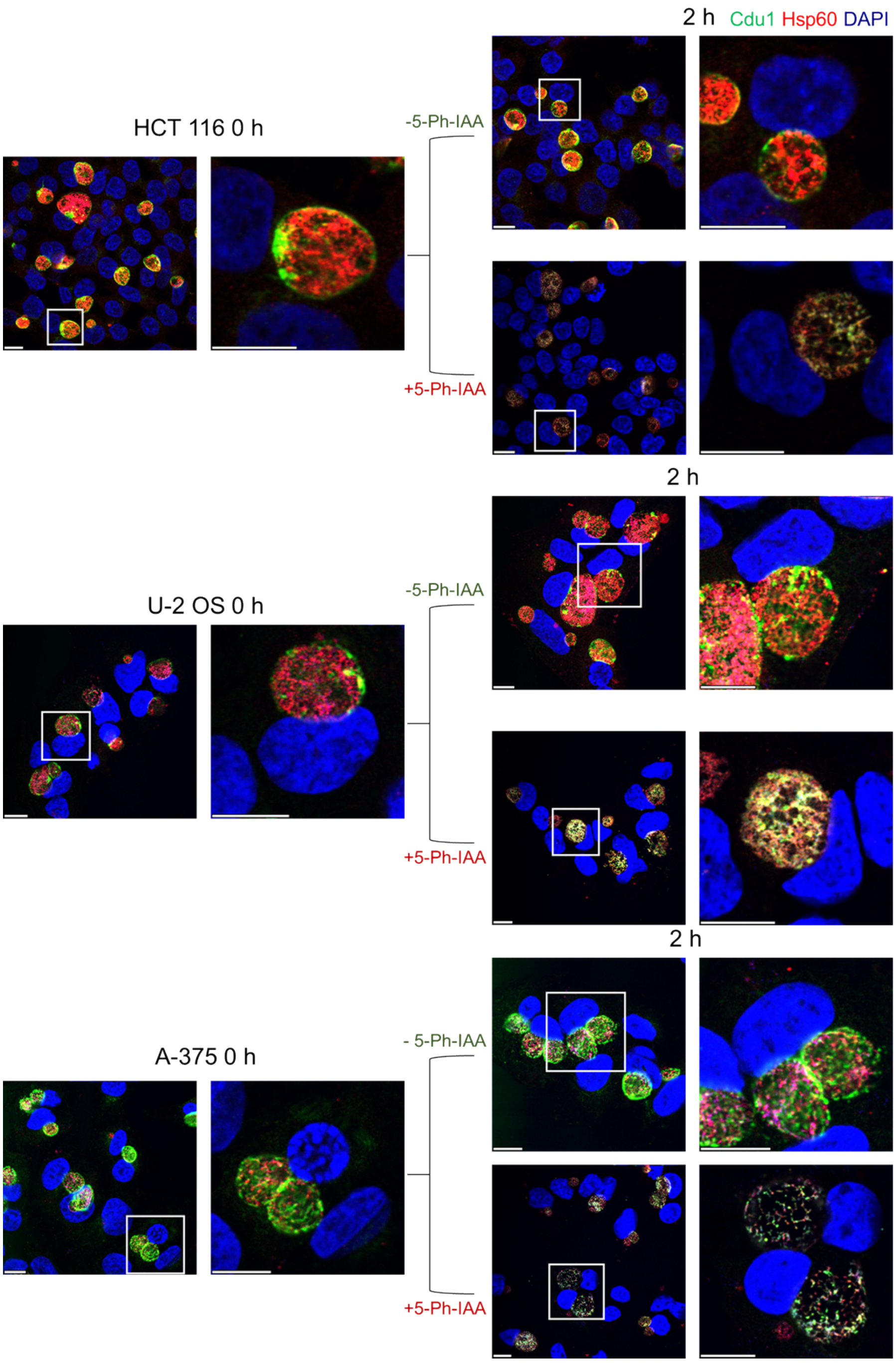

Supplementary Figure 6. Immunofluorescence microscopy confirms Cdu1 depletion dynamics in A-375, HCT 116 and U-2 OS cells. Cdu1 (green, anti-FLAG) was visualized with Chlamydia Hsp60 (red), to mark inclusions and DAPI (blue) to label bacterial and host DNA. The white square in the left panel marks the region enlarged in the right panel. Scale bar = 10 µm. All experiments were replicated ≥3 times with consistent results.

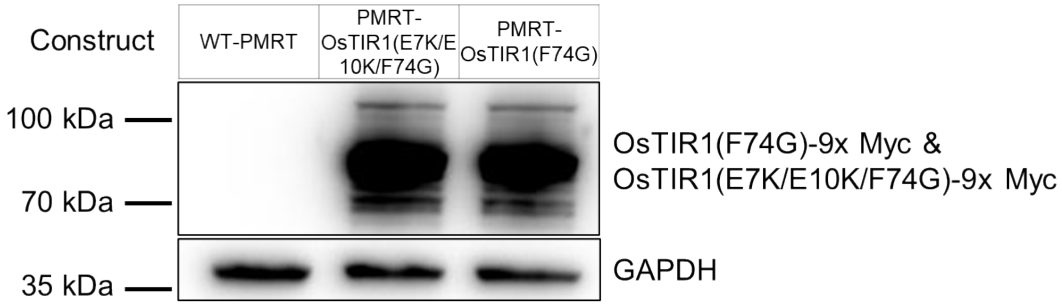

Supplementary Figure 7. Expression of OsTIR1(F74G) and OsTIR1(E7K/E10K/F74G) in PMRT cells. OsTIR1 mutants (anti-Myc) were monitored, with GAPDH as a loading control. All experiments were replicated ≥3 times with consistent results. Source data are provided as a Source Data file.

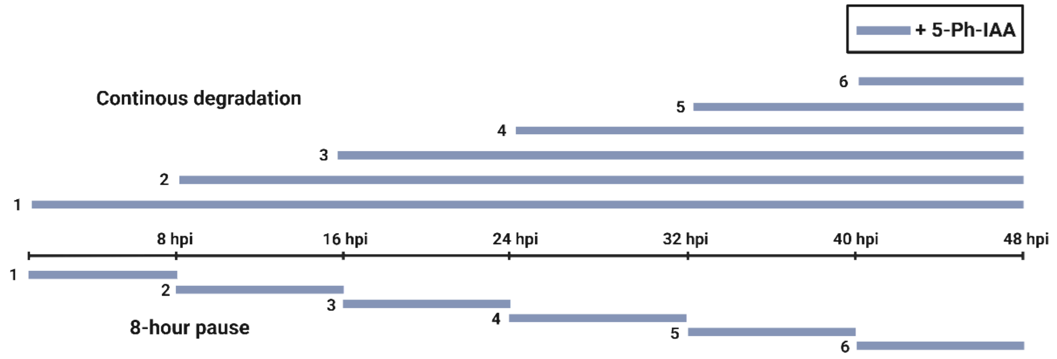

Supplementary Figure 8. **Schematic of temporal Ctr-AIDE-mediated Cdu1 degradation to evaluate its role in chlamydial growth.** The blue lines indicate periods of 1 μM 5-Ph-IAA treatment to induce Cdu1-mAID degradation. Bacterial growth and progeny-associated secondary infection burden were assessed at 48 hpi.

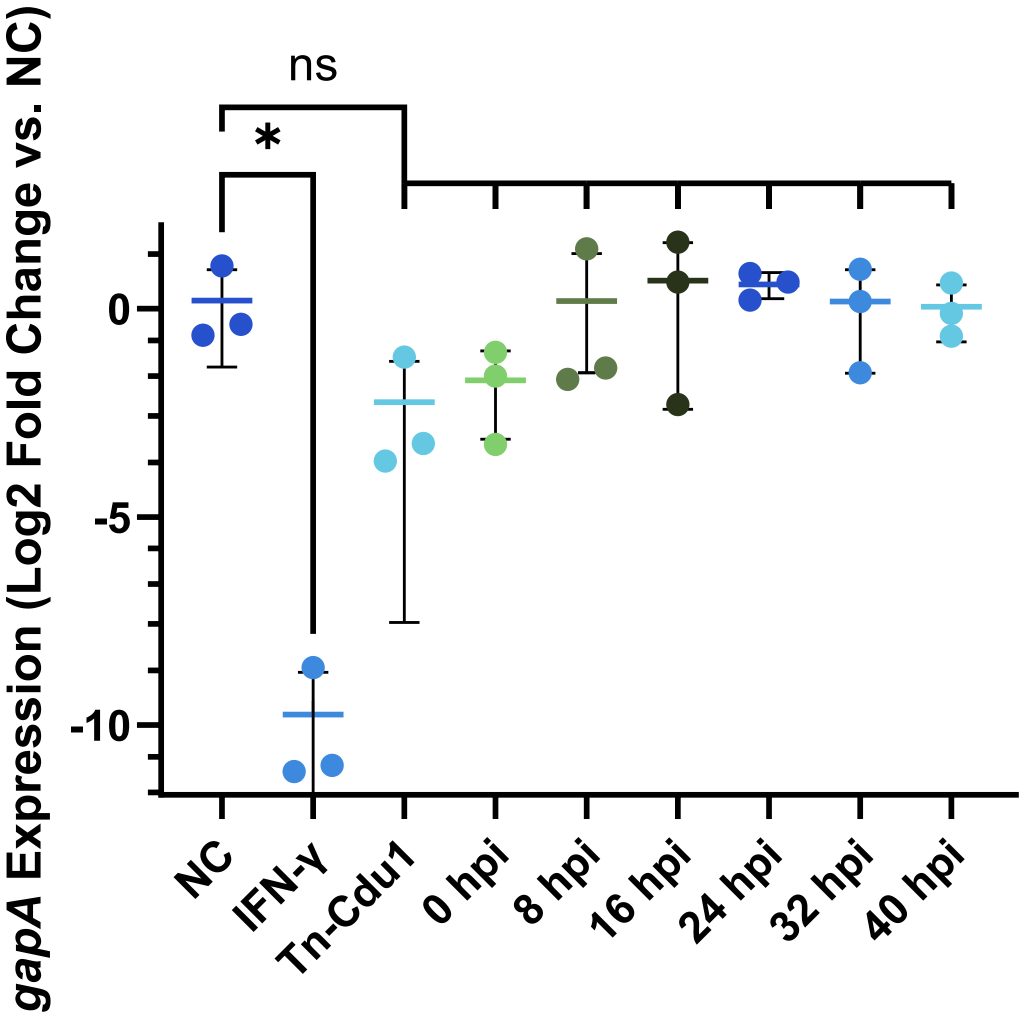

Supplementary Figure 9. Cdu1 depletion does not impair *Chlamydia* metabolic activity in HeLa cells. RT-qPCR analysis of *gapA* (glyceraldehyde-3-phosphate dehydrogenase, metabolic marker) and *rrs* (16S rRNA, load control) in Cdu1-mAID strains treated with 5-Ph-IAA at indicated timepoints to 48 hpi. IFN-γ (40-hour treatment, 50 U/mL) served as a positive control for metabolic inhibition, Tn-Cdu1 strain serves as a Cdu1 dysfunctional control. Data are normalized to DMSO-treated controls (NC) and shown as mean ± SD from three independent biological replicates (n = 3). Significance assessed by one-way ANOVA with Dunnett Multiple comparisons test (*p < 0.05; n.s., not significant, exact p values were provided in Supplementary Data 3). Experiments were replicated ≥3 times with consistent results. Source data are provided as a Source Data file.

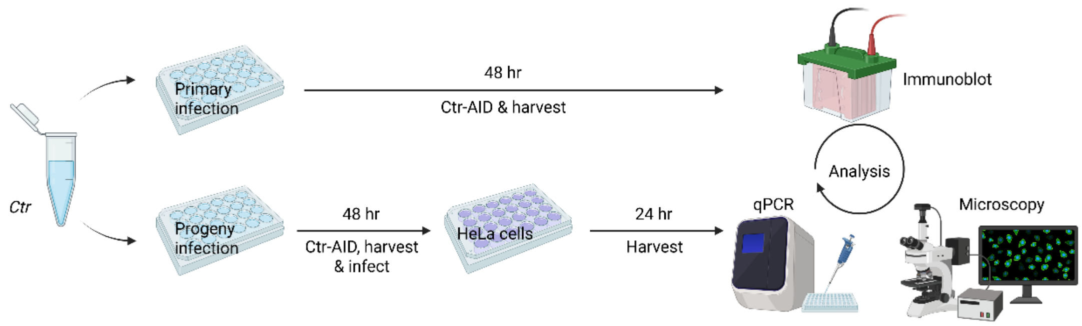

Supplementary Figure 10. Schematic representation of the primary and progeny infection assays. Created in BioRender. Zhang, H. (2026) https://BioRender.com/pb6d4qr

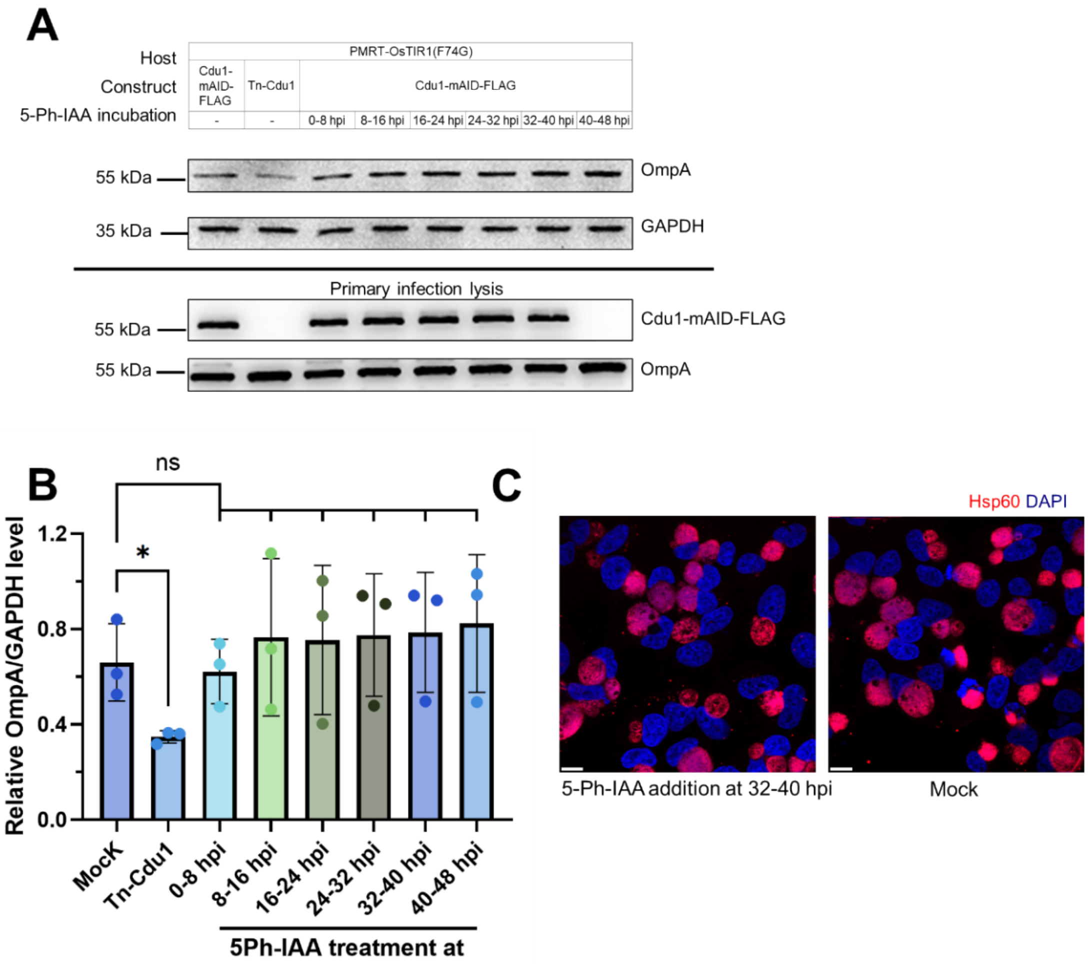

Supplementary Figure 11. Short-term Cdu1 depletion does not affect *C. trachomatis* progeny-associated secondary infection burden in primary cells. (A) Chlamydial lysates from primary infection (MOI=1, Cdu1 degraded at the indicated time points for 8 hours) were normalized by OmpA levels ('Primary infection lysis' part of the immunoblot image) and used to infect new batch of HeLa cells. 24 hpi, OmpA (secondary infection burden) and GAPDH (loading control) were analyzed via immunoblot. Tn-Cdu1 strain serves as a Cdu1 dysfunctional control. (B) Quantification of effects of 8 hours Cdu1 expression pause on progeny-associated secondary infection burden. OmpA levels were calculated (normalized to GAPDH, mean ± SD) from three replicates (n = 3, one representative in Panel A). Statistical significance determined by one-way ANOVA with Dunnett Multiple comparisons test (*$p < 0.05$; n.s., not significant, exact p values were provided in Supplementary Data 3). (C) Immunofluorescence microscopy reveals no reduction in secondary infection. HeLa cells were infected with progeny derived from Cdu1-degraded (degraded from 32 to 40 hpi) or control *Chlamydia*. Images were captured at 24 hpi. Inclusions marked by Hsp60 (red) and DNA (DAPI, blue) marking bacterial and host nuclei; scale bar = 10 µm. All experiments were replicated ≥3 times with consistent results. Source data are provided as a Source Data file.

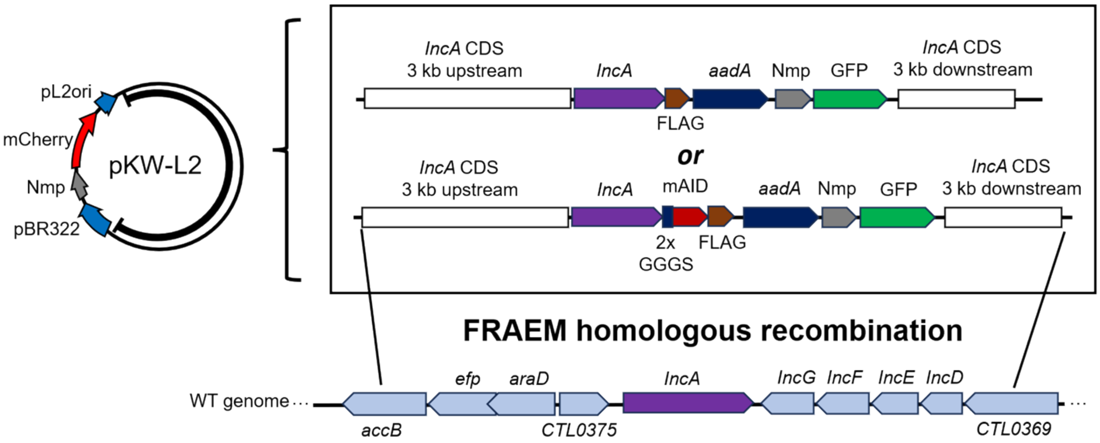

Supplementary Figure 12. Plasmids used in FRAEM homologous recombination for IncA.

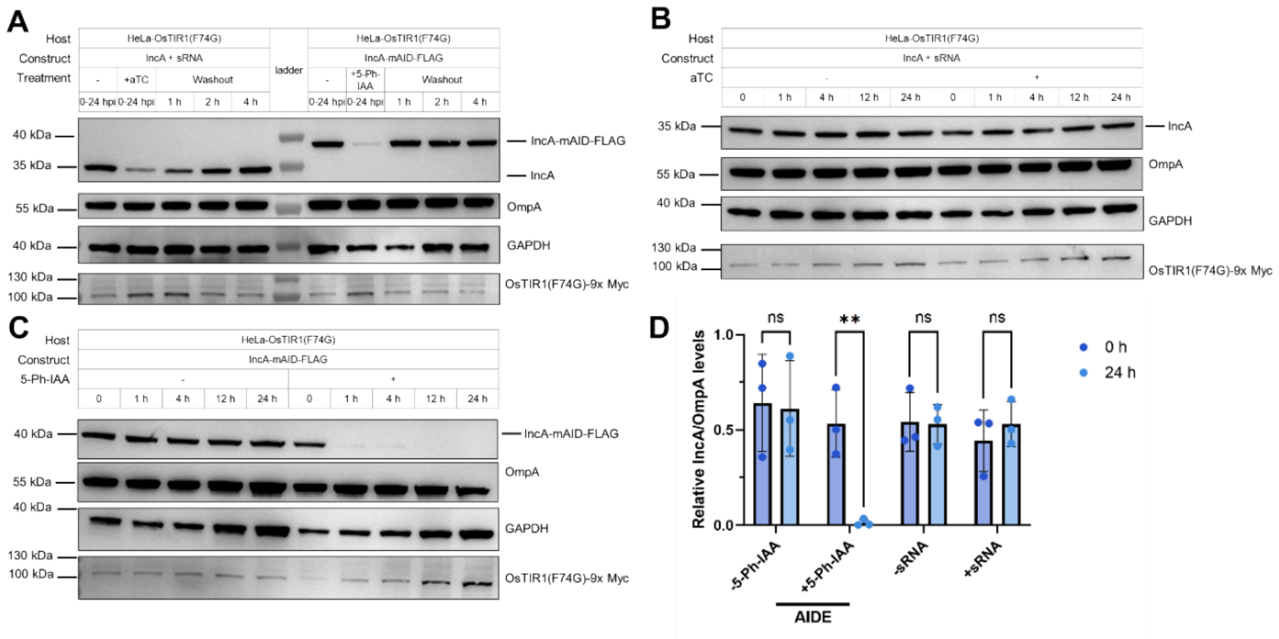

Supplementary Figure 13. Comparison of Ctr-AIDE-mediated and sRNA-based IncA depletion in *C. trachomatis*. (A) Comparison of sRNA silencing and Ctr-AIDE-mediated regulation of IncA expression from 0–24 hpi. Both approaches reduced IncA levels upon early life cycle inhibition. HeLa cells expressing OsTIR1(F74G) were infected with *C. trachomatis* expressing Cdu1-mAID or engineered sRNA targeting IncA (MOI = 1). Cells were treated with 1 μM 5-Ph-IAA or 200 ng/mL anhydrotetracycline (aTC; to induce sRNA expression), then washed and incubated in inducer-free medium for the indicated times. Cdu1 (anti-FLAG) and OsTIR1(F74G) (anti-Myc) levels were monitored, with Chlamydia OmpA as a loading control and host GAPDH as a cellular control. (B) sRNA silencing initiated at 24 hpi failed to deplete IncA. At 24 hpi, cells were treated with 200 ng/mL aTC (to induce sRNA expression) or DMSO for the indicated times. All experiments were replicated ≥3 times with consistent results. (C) Ctr-AIDE enabled rapid and efficient depletion of IncA. At 24 hpi, cells were treated with 1 μM 5-Ph-IAA or DMSO for the indicated times. (D) Quantification and comparation of IncA degradation efficiency by Ctr-AIDE and sRNA silencing initiated at 24 hpi. Quantification shows IncA levels normalized to OmpA (mean ± SD; n = 3 biological replicates). Significance assessed by two-tailed paired t-test (**$p < 0.01$; n.s., not significant, exact p values were provided in Supplementary Data 3). All experiments were replicated ≥3 times with consistent results. Source data are provided as a Source Data file.

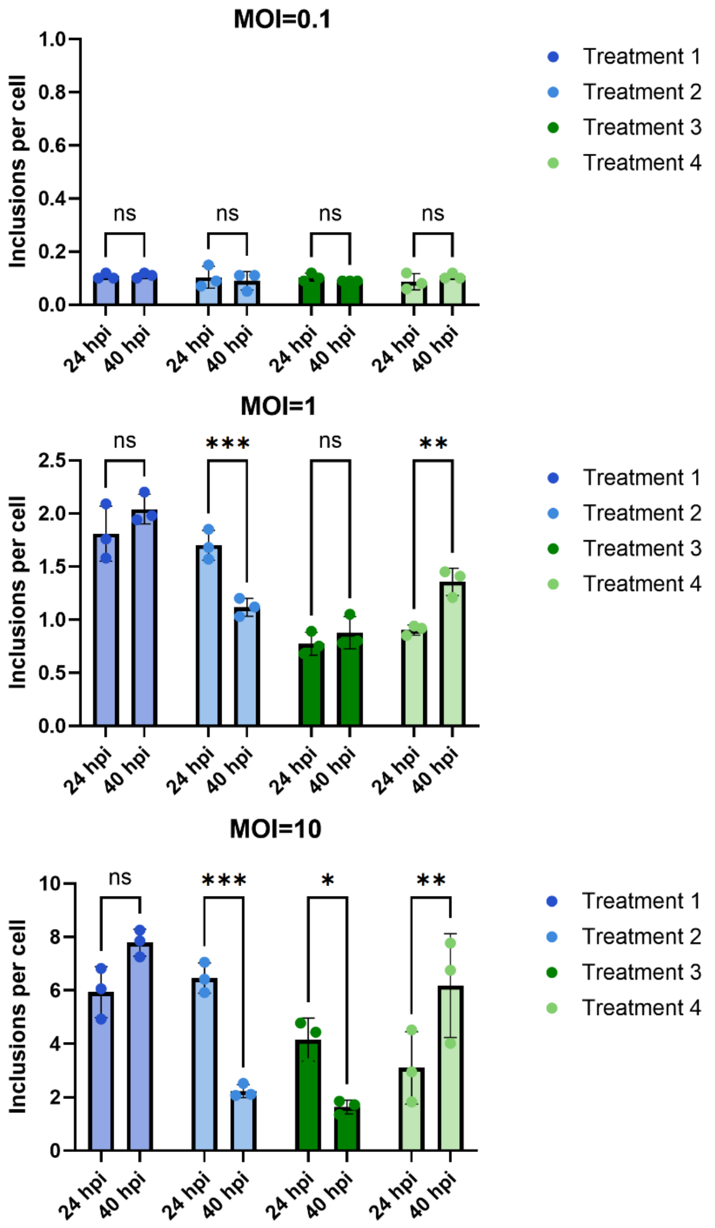

Supplementary Figure 14. The IncA-dependent multi-inclusion phenotype is MOI dependent. HeLa cells expressing OsTIR1(F74G) were infected with the IncA-mAID strain and subjected to Treatments 1–4 as outlined in Fig. 6B at MOI = 0.1, 1, or 10. Inclusion number per infected cell was quantified at 24 and 40 hpi. Data are shown as mean ± SD from 3 independent biological replicates (n = 3). Statistical significance was assessed by two-tailed paired t-test (***p < 0.001, **p < 0.01, *p < 0.05; n.s., not significant, exact p values were provided in Supplementary Data 3). All experiments were replicated ≥3 times with consistent results. Source data are provided as a Source Data file.

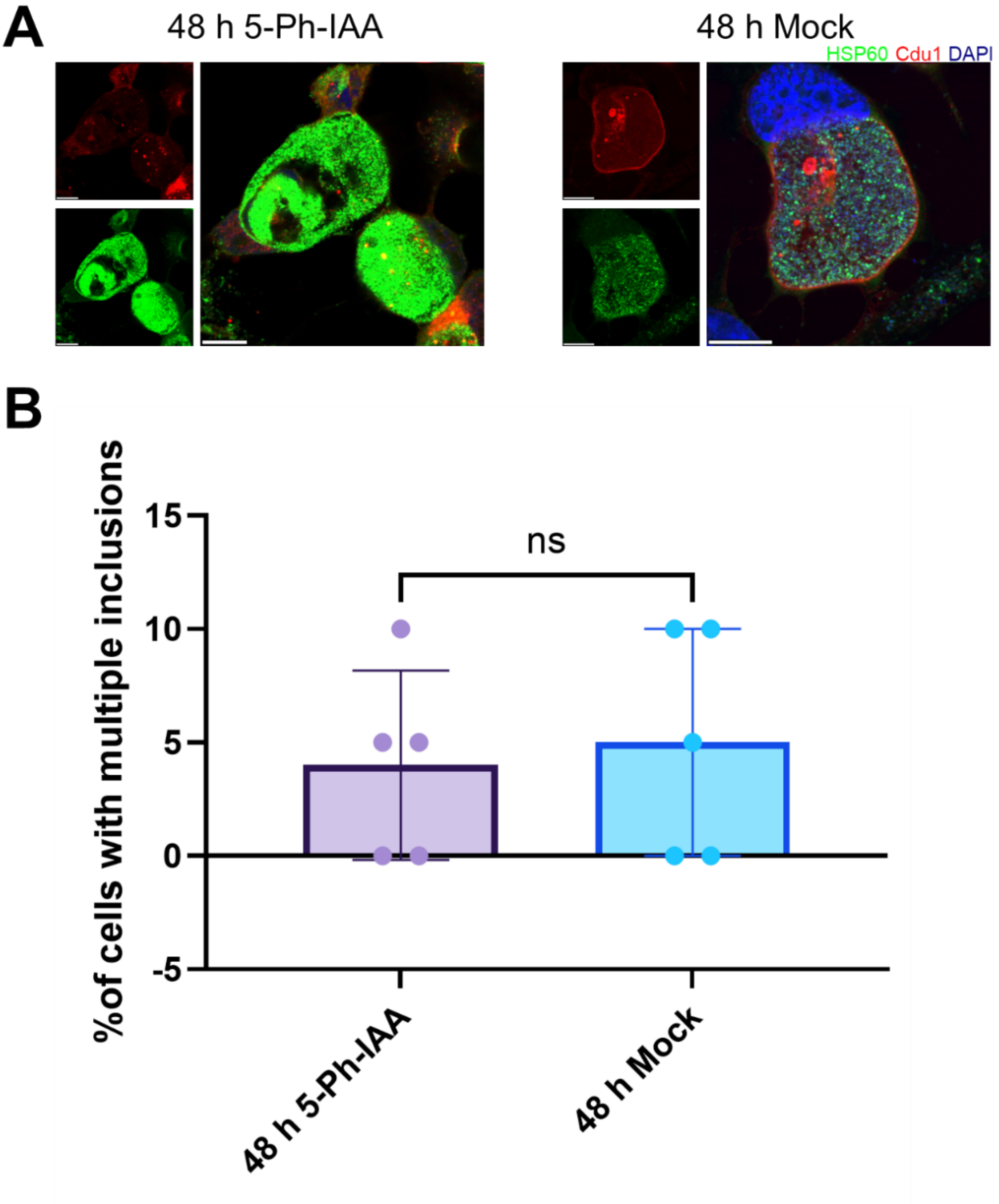

Supplementary Figure 15. Prolonged Cdu1 degradation by Ctr-AIDE did not increase the fraction of cells containing multiple inclusions. (A) HeLa cells expressing OsTIR1(F74G) infected with Cdu1-mAID strains (MOI=1) were treated with 5-Ph-IAA (Cdu1 degraded) or DMSO (Mock) from infection until 48 hpi. Both degraded and control samples show cells containing predominantly single inclusions. Cdu1 (red, anti-FLAG), inclusions (Hsp60, green), and DNA (DAPI, blue). Scale bar = 10 μm. (B) Quantification of host cells containing multiple intact inclusions (≥2) upon Cdu1 degradation. Data represents mean percentage ± SD from total 100 cells counted per condition across five independent biological replicates ($n = 5$). Significance assessed by two-tailed unpaired t-test (ns, not significant, exact p values were provided

in Supplementary Data 3). All experiments were replicated ≥3 times with consistent results. Source data are provided as a Source Data file.

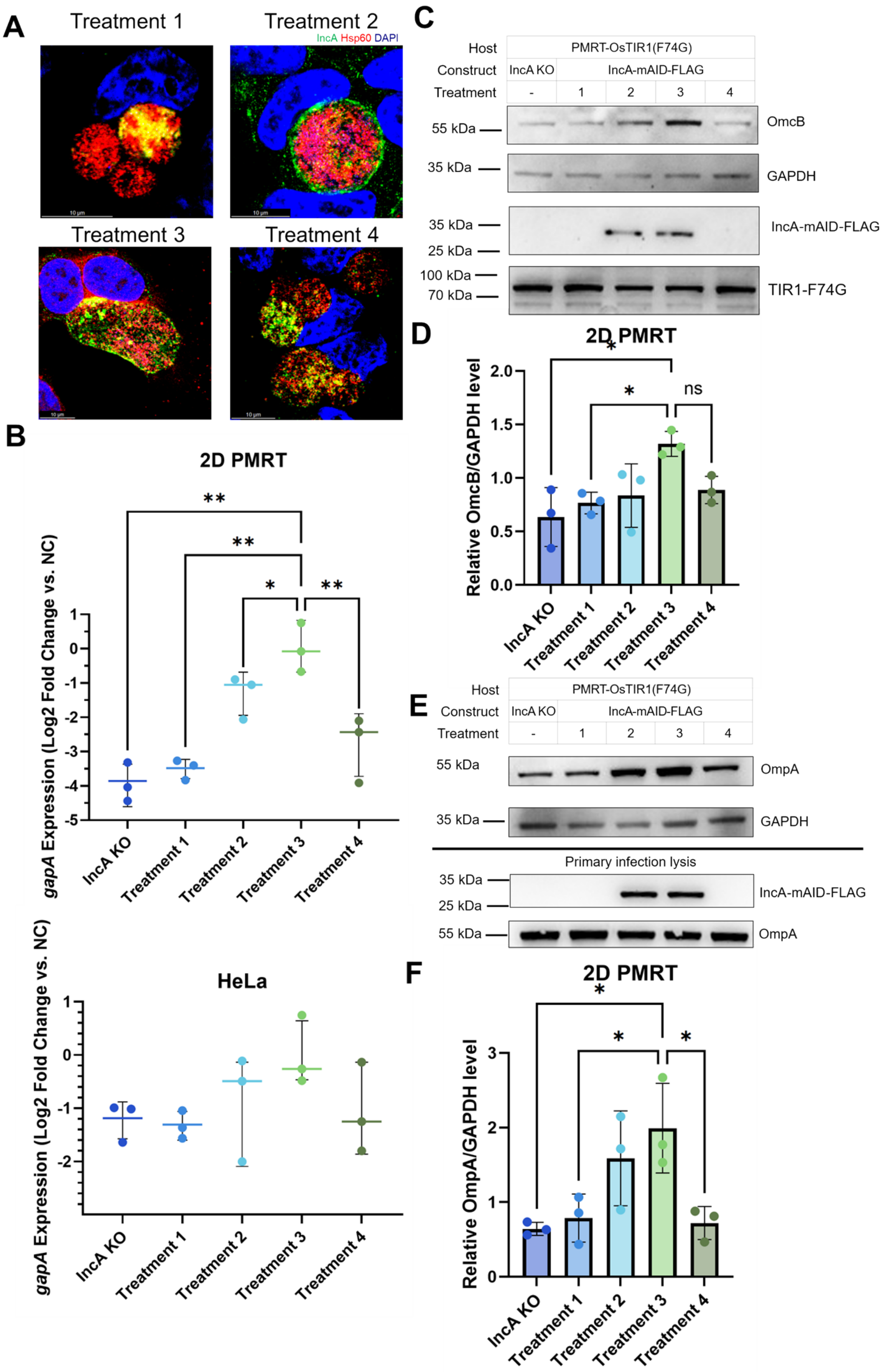

Supplementary Figure 16. Host cell–dependent effects of IncA degradation on chlamydial metabolism, differentiation, and progeny-associated secondary infection burden. (A) IncA degradation triggers multi-inclusions phenotype in primary

cells. PMRT cells expressing OsTIR1(F74G) were infected with IncA-mAID strains (MOI = 1) and subjected to Treatments 1–4 shown in Fig. 6B. Representative images were acquired at 40 hpi. IncA (green, anti-FLAG), inclusions (Hsp60, red), and DNA (DAPI, blue). Scale bar = 10 µm. (B) IncA depletion reduces *C. trachomatis* metabolic activity in primary cells (up) but not in HeLa cells (down). RT-qPCR analysis of gapA (metabolic marker) and rrs (16S rRNA; normalization control) at 40 hpi in IncA-mAID strains subjected to the Treatment 1-4. An IncA knockout strain (IncA KO) served as an additional control. Data from three independent replicates (mean ± SD, n = 3) were normalized to treatment 3 (constant IncA expression). Statistical analysis by one-way ANOVA with Dunnett's test (**$p < 0.01$, *$p < 0.05$; Non-significant comparisons (n.s.) are not shown). (C) IncA depletion disrupts RB-to-EB redifferentiation in primary cells. Immunoblot analysis of OmcB (EB marker) in IncA-mAID–infected primary cells (MOI = 1; 40 hpi) subjected to the indicated treatments. IncA (anti-FLAG) and OsTIR1(F74G) (anti-Myc) blots confirm protein degradation; GAPDH serves as a loading control. (D) Quantification of OmcB levels normalized to GAPDH from three independent experiments (mean ± SD, n = 3; representative blots shown in C). Statistical significance was assessed by one-way ANOVA with Dunnett's multiple comparisons test (*$p < 0.05$). Non-significant comparisons (n.s.) are not shown, except for the comparison between treatments 3 and 4. (E) IncA loss reduces progeny-associated secondary infection burden in primary cells. Lysates from primary infections (MOI = 1; indicated treatments until 40 hpi) were normalized based on OmpA levels in the primary infection (lower panel) and used to infect fresh HeLa cells. (F) Quantification of OmpA abundance in secondary infections at 24 hpi. OmpA levels were normalized to GAPDH and are shown as mean ± SD (n = 3; representative blots shown in E). Statistical analysis was performed using one-way ANOVA with Dunnett's multiple comparisons test (Non-significant comparisons are not shown). Exact p values were provided in Supplementary Data 3. All experiments were replicated ≥3 times with consistent results. Source data are provided as a Source Data file.

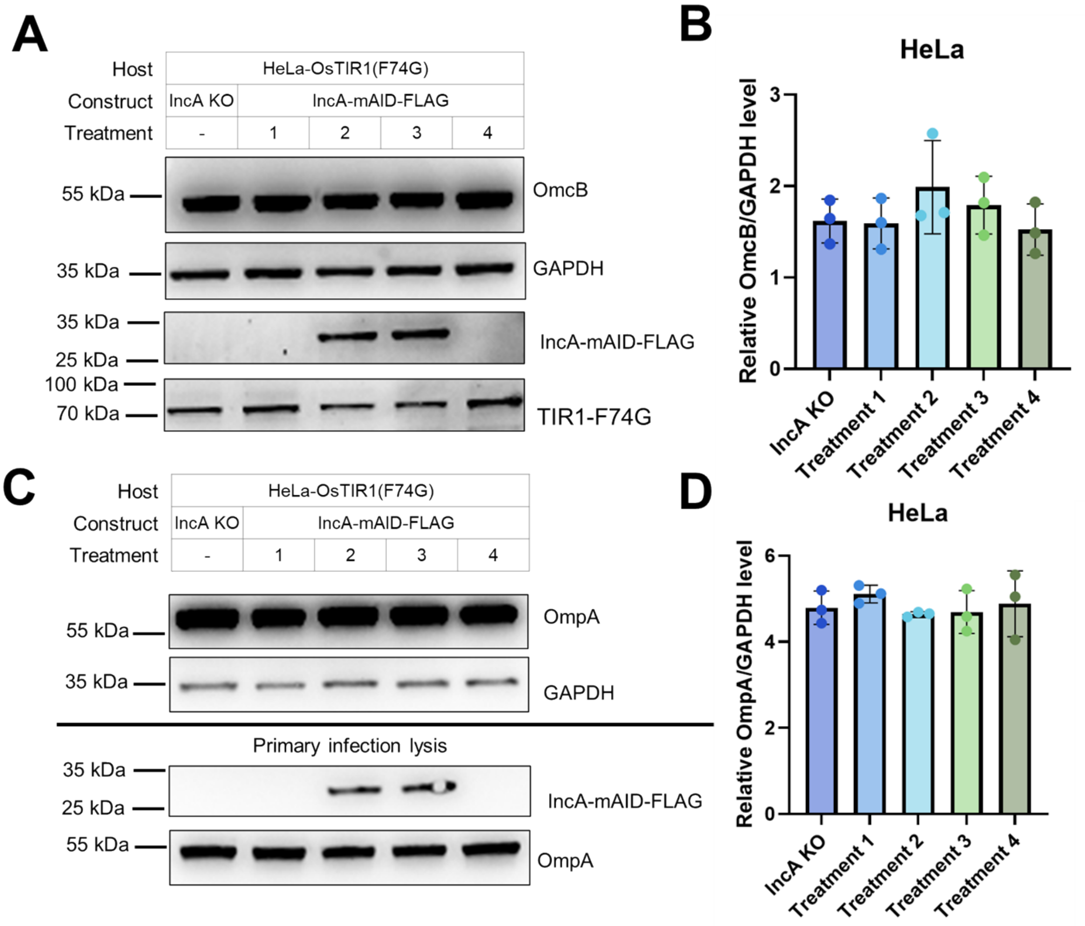

Supplementary Figure 17. Effects of IncA degradation on chlamydial differentiation, and progeny-associated secondary infection burden in HeLa cells. (A) IncA depletion does not impair RB-to-EB redifferentiation in HeLa cells. Immunoblot analysis of OmcB in IncA-mAID–infected HeLa cells subjected to the indicated treatments, with degradation and loading controls as in supplementary figure 15. (B) Quantification of OmcB levels normalized to GAPDH from three independent experiments (n = 3, mean ± SD; representative blots shown in A). Statistical significance was assessed by one-way ANOVA with Dunnett's multiple comparisons test, (Non-significant comparisons are not shown, exact p values were provided in Supplementary Data 3). (C) IncA loss does not reduce progeny-associated secondary infection burden in HeLa cells. Lysates from primary HeLa cell infections (MOI = 1; indicated treatments until 40 hpi) were normalized by primary-infection OmpA levels and used to infect fresh HeLa cells. (D) Quantification of OmpA abundance in secondary infections at 24 hpi. OmpA levels were normalized to GAPDH and are shown as mean ± SD (n = 3; representative blots shown in C). Statistical analysis was performed using one-way ANOVA with Dunnett's multiple comparisons test (Non-significant comparisons are not shown, exact p values were provided in Supplementary Data 3). All experiments were replicated ≥3 times with consistent results. Source data are provided as a Source Data file.

Supplemental References

1. Gordon, F.B. & Quan, A.L. ISOLATION OF THE TRACHOMA AGENT IN CELL CULTURE. *Proc Soc Exp Biol Med* **118**, 354-359 (1965).
2. Yee, C., Krishnan-Hewlett, I., Baker, C.C., Schlegel, R. & Howley, P.M. Presence and expression of human papillomavirus sequences in human cervical carcinoma cell lines. *Am J Pathol* **119**, 361-366 (1985).
3. DuBridge, R.B. et al. Analysis of mutation in human cells by using an Epstein-Barr virus shuttle system. *Mol Cell Biol* **7**, 379-387 (1987).
4. Giard, D.J. et al. In vitro cultivation of human tumors: establishment of cell lines derived from a series of solid tumors. *J Natl Cancer Inst* **51**, 1417-1423 (1973).
5. Schroy, P.C., 3rd et al. Detection of p21ras mutations in colorectal adenomas and carcinomas by enzyme-linked immunosorbent assay. *Cancer* **76**, 201-209 (1995).
6. Heldin, C.H. et al. A human osteosarcoma cell line secretes a growth factor structurally related to a homodimer of PDGF A-chains. *Nature* **319**, 511-514 (1986).
7. Fields, K.A., Bodero, M.D., Scanlon, K.R., Jewett, T.J. & Wolf, K. A Minimal Replicon Enables Efficacious, Species-Specific Gene Deletion in Chlamydia and Extension of Gene Knockout Studies to the Animal Model of Infection Using Chlamydia muridarum. *Infect Immun* **90**, e0045322 (2022).
8. Ehses, J. et al. Development of an sRNA-mediated conditional knockdown system for Chlamydia trachomatis. *mBio* **16**, e0254524 (2025).
9. Schachter, J. et al. Lymphogranuloma venereum. I. Comparison of the Frei test, complement fixation test, and isolation of the agent. *J Infect Dis* **120**, 372-375 (1969).
