# Supplementary material for: Minute-scale control of ubiquitin-mediated degradation reveals dynamics of bacterial secreted effector-functions
Source: Nat Commun. 2026 May 18;17:4420. doi: 10.1038/s41467-026-73213-x (PMC13183884; doi:10.1038/s41467-026-73213-x)
Supplement: Supplementary file 2 — Description of Additional Supplementary Information [file 41467_2026_73213_MOESM2_ESM.pdf]

Description of Additional Supplementary Files

File Name: Supplementary Data 1

Description: DNA sequences of plasmids used in this study. Complete plasmid sequences for all constructs generated in this work are provided in Excel format.

File Name: Supplementary Data 2

Description: Primers used in this study. Complete primers sequences used in this work are provided in Excel format.

File Name: Supplementary Data 3

Description: **Exact p values of statistical test in this study.** Exact p values and their corresponding significance summaries for all comparisons in this study are provided in Excel format.

File Name: Supplementary Movie 1

Description: Continuous IncA depletion results in a persistent multiinclusion phenotype. Inclusions are shown in green (GFP). Time stamps are indicated at the top left. Scale bar, 10 μm.

File Name: Supplementary Movie 2

Description: Continuous IncA depletion results in a persistent multiinclusion phenotype. Inclusions are shown in green (GFP). Time stamps are indicated at the top left. Scale bar, 10 μm.

File Name: Supplementary Movie 3

Description: Continuous IncA depletion results in a persistent multiinclusion phenotype. Inclusions are shown in green (GFP). Time stamps are indicated at the top left. Scale bar, 10 μm.

File Name: Supplementary Movie 4

Description: IncA reexpression promotes inclusion fusion. Inclusions are shown in green (GFP). Time stamps are indicated at the top left. Scale bar, 10 μm.

File Name: Supplementary Movie 5

Description: IncA reexpression promotes inclusion fusion. Inclusions are shown in green (GFP). Time stamps are indicated at the top left. Scale bar, 10 μm.

File Name: Supplementary Movie 6

Description: IncA reexpression promotes inclusion fusion. Inclusions are shown in green (GFP). Time stamps are indicated at the top left. Scale bar, 10 μm.

File Name: Supplementary Movie 7

Description: Continuous IncA expression is associated predominantly with single inclusions. Inclusions are shown in green (GFP). Time stamps are indicated at the top left. Scale bar, 10 μm.

File Name: Supplementary Movie 8

Description: Continuous IncA expression is associated predominantly with single inclusions. Inclusions are shown in green (GFP). Time stamps are indicated at the top left. Scale bar, 10 μm.

File Name: Supplementary Movie 9

Description: Continuous IncA expression is associated predominantly with single inclusions. Inclusions are shown in green (GFP). Time stamps are indicated at the top left. Scale bar, 10 μm.

File Name: Supplementary Movie 10

Description: Acute IncA degradation leads to inclusion fission. Inclusions are shown in green (GFP). Time stamps are indicated at the top left. Scale bar, 10 μm.

File Name: Supplementary Movie 11

Description: Acute IncA degradation leads to inclusion fission. Inclusions are shown in green (GFP). Time stamps are indicated at the top left. Scale bar, 10 μm.

File Name: Supplementary Movie 12

Description: Acute IncA degradation leads to inclusion fission. Inclusions are shown in green (GFP). Time stamps are indicated at the top left. Scale bar, 10 μm.
