# Supplementary material for: Minute-scale control of ubiquitin-mediated degradation reveals dynamics of bacterial secreted effector-functions
Source: Nat Commun. 2026 May 18;17:4420. doi: 10.1038/s41467-026-73213-x (PMC13183884; doi:10.1038/s41467-026-73213-x)
Supplement: Supplementary file 18 — Reporting Summary [file 41467_2026_73213_MOESM18_ESM.pdf]

nature portfolio

Corresponding author(s): Thomas Rudel

Last updated by author(s): Apr 10, 2026

Reporting Summary

Nature Portfolio wishes to improve the reproducibility of the work that we publish. This form provides structure for consistency and transparency in reporting. For further information on Nature Portfolio policies, see our Editorial Policies and the Editorial Policy Checklist.

Statistics

For all statistical analyses, confirm that the following items are present in the figure legend, table legend, main text, or Methods section.

n/a	Confirmed	
☐	☒	The exact sample size (n) for each experimental group/condition, given as a discrete number and unit of measurement
☐	☒	A statement on whether measurements were taken from distinct samples or whether the same sample was measured repeatedly
☐	☒	The statistical test(s) used AND whether they are one- or two-sided<br>*Only common tests should be described solely by name; describe more complex techniques in the Methods section.*
☐	☒	A description of all covariates tested
☐	☒	A description of any assumptions or corrections, such as tests of normality and adjustment for multiple comparisons
☐	☒	A full description of the statistical parameters including central tendency (e.g. means) or other basic estimates (e.g. regression coefficient) AND variation (e.g. standard deviation) or associated estimates of uncertainty (e.g. confidence intervals)
☐	☒	For null hypothesis testing, the test statistic (e.g. F, t, r) with confidence intervals, effect sizes, degrees of freedom and P value noted<br>*Give P values as exact values whenever suitable.*
☒	☐	For Bayesian analysis, information on the choice of priors and Markov chain Monte Carlo settings
☒	☐	For hierarchical and complex designs, identification of the appropriate level for tests and full reporting of outcomes
☒	☐	Estimates of effect sizes (e.g. Cohen's d, Pearson's r), indicating how they were calculated

Our web collection on statistics for biologists contains articles on many of the points above.

Software and code

Policy information about availability of computer code

Data collection	ImageJ (v1.53), Leica LAS X software (3.10.0).
Data analysis	GraphPad Prism (v10.4.0)

For manuscripts utilizing custom algorithms or software that are central to the research but not yet described in published literature, software must be made available to editors and reviewers. We strongly encourage code deposition in a community repository (e.g. GitHub). See the Nature Portfolio guidelines for submitting code & software for further information.

Data

Policy information about availability of data

All manuscripts must include a data availability statement. This statement should provide the following information, where applicable:

- Accession codes, unique identifiers, or web links for publicly available datasets
- A description of any restrictions on data availability
- For clinical datasets or third party data, please ensure that the statement adheres to our policy

The authors declare that the data supporting the findings of this study are available within the paper and its Souces data file. in addition, We the raw data files exported directly from Leica LAS X, ImageJ, and the StepOnePlus system have been deposited in Figshare, and the corresponding dataset has been included in the reference list.

Research involving human participants, their data, or biological material

Policy information about studies with human participants or human data. See also policy information about sex, gender (identity/presentation), and sexual orientation and race, ethnicity and racism.

Reporting on sex and gender	*Use the terms sex (biological attribute) and gender (shaped by social and cultural circumstances) carefully in order to avoid confusing both terms. Indicate if findings apply to only one sex or gender; describe whether sex and gender were considered in study design; whether sex and/or gender was determined based on self-reporting or assigned and methods used. Provide in the source data disaggregated sex and gender data, where this information has been collected, and if consent has been obtained for sharing of individual-level data; provide overall numbers in this Reporting Summary. Please state if this information has not been collected. Report sex- and gender-based analyses where performed, justify reasons for lack of sex- and gender-based analysis.*
Reporting on race, ethnicity, or other socially relevant groupings	*Please specify the socially constructed or socially relevant categorization variable(s) used in your manuscript and explain why they were used. Please note that such variables should not be used as proxies for other socially constructed/relevant variables (for example, race or ethnicity should not be used as a proxy for socioeconomic status). Provide clear definitions of the relevant terms used, how they were provided (by the participants/respondents, the researchers, or third parties), and the method(s) used to classify people into the different categories (e.g. self-report, census or administrative data, social media data, etc.) Please provide details about how you controlled for confounding variables in your analyses.*
Population characteristics	*Describe the covariate-relevant population characteristics of the human research participants (e.g. age, genotypic information, past and current diagnosis and treatment categories). If you filled out the behavioural & social sciences study design questions and have nothing to add here, write "See above."*
Recruitment	*Describe how participants were recruited. Outline any potential self-selection bias or other biases that may be present and how these are likely to impact results.*
Ethics oversight	*Identify the organization(s) that approved the study protocol.*

Note that full information on the approval of the study protocol must also be provided in the manuscript.

Field-specific reporting

Please select the one below that is the best fit for your research. If you are not sure, read the appropriate sections before making your selection.

☒ Life sciences ☐ Behavioural & social sciences ☐ Ecological, evolutionary & environmental sciences

For a reference copy of the document with all sections, see nature.com/documents/nr-reporting-summary-flat.pdf

Life sciences study design

All studies must disclose on these points even when the disclosure is negative.

Sample size	Sample sizes were chosen based on standard practice in the field and on the need to ensure reproducibility while maintaining feasibility for the respective assays. For immunoblot and RT-qPCR analyses, three independent biological replicates were used to capture biological variability and ensure reproducibility. For p62 inclusion quantification, 500 inclusions across five independent biological replicates were analyzed to reduce sampling bias and provide robust measurements. For quantification of the percentage of cells containing multiple inclusions during IncA depletion, 200 infected cells across five independent biological replicates were analyzed to obtain representative measurements while maintaining practical feasibility. For the corresponding analysis during Cdu1 depletion, 100 infected cells across five independent biological replicates were analyzed for the same reason. For quantification of inclusion number per infected cell across different MOIs during IncA degradation, 100 infected cells across three independent biological replicates were analyzed to obtain representative measurements while balancing feasibility.
Data exclusions	All collected data were included; no exclusions were made.
Replication	All experiments were independently repeated at least three times, and consistent results were obtained across replicates, confirming reproducibility.
Randomization	The experiments were not randomized.
Blinding	The investigators were not blinded to allocation during experiments and outcome assessment

Reporting for specific materials, systems and methods

We require information from authors about some types of materials, experimental systems and methods used in many studies. Here, indicate whether each material, system or method listed is relevant to your study. If you are not sure if a list item applies to your research, read the appropriate section before selecting a response.

Materials & experimental systems

n/a	Involved in the study
	☒ Antibodies
	☒ Eukaryotic cell lines
☒	Palaeontology and archaeology
	☒ Animals and other organisms
☒	Clinical data
☒	Dual use research of concern
☒	Plants

Methods

n/a	Involved in the study
☒	ChIP-seq
☒	Flow cytometry
☒	MRI-based neuroimaging

Antibodies

Antibodies used

anti-cHSP60 (A57-B9), 1:200 (IF) 1:50 (ExM), Primary antibody, Santa Cruz, #sc-57840
anti-p62, 1:200 (IF), Primary antibody, Santa Cruz, #sc-28359
anti-OmcB, 1:1000 (WB), Primary antibody, Invitrogen, #PA5-117552
anti-ompA, 1:500 (WB), Primary antibody, Invitrogen, #PA5-117609
anti-IncA 1:100 (WB) Primary antibody, self-made
anti-Cdu1 1:100 (WB) Primary antibody, self-made
anti-DDDDK tag, 1:500 (IF) 1:200 (ExM), Primary antibody, Abcam, #ab205606
Alexa Fluor™ Plus 488, 1:300 (IF), Secondary antibody, Invitrogen, #A32723
Alexa Fluor™ Plus 555, 1:300 (IF), Secondary antibody, Invitrogen, #A32732
anti-Rabbit IgG (H+L), HRP 1:50000 (WB), Secondary antibody, Invitrogen, #31460
anti-Mouse IgG (H+L), HRP 1:50000 (WB), Secondary antibody, Invitrogen, #31430
anti-alpha Tubulin HRP, 1:50000 (WB), Hrp preconjugated antibodies, Abcam, #ab185067
anti-Myc HRP, 1:1000 (WB), Hrp preconjugated antibodies, Abclonal, #AE026
anti-DDDDK-Tag HRP, 1:5000 (WB), Hrp preconjugated antibodies, Abclonal, #AE095
anti-GAPDH HRP, 1:25000 (WB), Hrp preconjugated antibodies, Abclonal, #19056
anti-β-Actin, 1:25000 (WB), Hrp preconjugated antibodies, Abclonal, # AC028

Validation

anti-cHSP60, recently used in PMID: 39154341.
anti-p62, Western blot analysis of SQSTM1 expression in SK-LMS-1 (A) and HeLa (B) whole cell lysates shown in manufacturer's website. Recently used in PMID: 39154341.
anti-OmcB, Positive WB detected in Recombinant protein as shown in manufacturer's website.
anti-ompA, Positive WB detected in Recombinant protein as shown in manufacturer's website.
anti-IncA & anti-Cdu1, vaildated with whole Chlamydial lysis.
anti-DDDDK tag, Abcam have tested this species and application combination of WB and IF and it works as shown in manufacturer's website.
Alexa Fluor™ Plus 488 and Alexa Fluor™ Plus 555, Invitrogen tested them working for IF as shown in manufacturer's website.
anti-Rabbit IgG (H+L) and anti-Mouse IgG (H+L), Invitrogen tested them working for WB as shown in manufacturer's website.
anti-alpha Tubulin HRP, Positive WB detected in Recombinant protein as shown in manufacturer's website.
anti-Myc HRP, validated with Western blot analysis of lysates from cells transfected with NLK-Myc-Tag, as shown in manufacturer's datasheet.
anti-DDDDK-Tag HRP, validated with Western blot analysis of lysates from cells transfected with GSK3B-C Protein, as shown in manufacturer's datasheet.
anti-GAPDH HRP, validated with Western blot analysis of various cell lysates, as shown in manufacturer's datasheet.
anti-β-Actin, validated with Western blot analysis of various cell lysates, as shown in manufacturer's datasheet.

Eukaryotic cell lines

Policy information about cell lines and Sex and Gender in Research

Cell line source(s)

McCoy, ATCC CRL-1696
HeLa 229, ATCC CCL-2.1
HEK293T, ATCC CRL-3216
A-375, ATCC CRL-1619
HCT 116, ATCC CCL-247
U-2 OS, ATCC HTB-96
Primary Murine Reproductive Tract cells, extracted from the entire reproductive tract of 3 adult (7-8 weeks old) female C57BL/6J mice.

Authentication

Human cell lines were authenticated by STR profiling and routinely tested for mycoplasma contamination. Primary murine reproductive tract cells were derived from organoids established from whole reproductive tract tissue of adult female C57BL/6J mice as described in the Methods and were not subject to STR authentication.

Mycoplasma contamination

We confirmed that all cell lines were tested negative for mycoplasma contamination.

Commonly misidentified lines (See ICLAC register)	*Name any commonly misidentified cell lines used in the study and provide a rationale for their use.*

Animals and other research organisms

Policy information about studies involving animals; ARRIVE guidelines recommended for reporting animal research, and Sex and Gender in Research

Laboratory animals	C57BL/6J mice (Mus musculus) were originally obtained from Charles River and subsequently bred and maintained in-house at the animal facility of the Biocenter, Julius-Maximilians-Universität Würzburg, Würzburg, Germany. Mice were housed in individually ventilated cages (IVCs) with a 12 h light/12 h dark cycle, at an ambient temperature of 23±2°C and relative humidity of 50–60%, with food and water provided ad libitum.<br>In this study, mice were used solely as a source of tissue for organoid generation. Female C57BL/6J mice aged 8–9 weeks (n = 3) were used for isolation of female reproductive tract tissues for organoid culture. Mice were euthanized by cervical dislocation prior to tissue collection.
Wild animals	This study did not involve wild animals.
Reporting on sex	Only female mice were used, as the study relies on organoids derived from the female reproductive tract, which is the relevant tissue for Chlamydia infection.
Field-collected samples	This study did not involve samples collected from the field
Ethics oversight	All procedures involving animals were conducted in accordance with institutional and national guidelines and were approved by the Regierung von Unterfranken, Würzburg, Germany (license number: TV-AZ 55.2.2-2532-2-762).

Note that full information on the approval of the study protocol must also be provided in the manuscript.

Plants

Seed stocks	*Report on the source of all seed stocks or other plant material used. If applicable, state the seed stock centre and catalogue number. If plant specimens were collected from the field, describe the collection location, date and sampling procedures.*
Novel plant genotypes	*Describe the methods by which all novel plant genotypes were produced. This includes those generated by transgenic approaches, gene editing, chemical/radiation-based mutagenesis and hybridization. For transgenic lines, describe the transformation method, the number of independent lines analyzed and the generation upon which experiments were performed. For gene-edited lines, describe the editor used, the endogenous sequence targeted for editing, the targeting guide RNA sequence (if applicable) and how the editor was applied.*
Authentication	*Describe any authentication procedures for each seed stock used or novel genotype generated. Describe any experiments used to assess the effect of a mutation and, where applicable, how potential secondary effects (e.g. second site T-DNA insertions, mosiacism, off-target gene editing) were examined.*
